# Local Application of Krill Oil Accelerates the Healing of Artificially Created Wounds in Diabetic Mice

**DOI:** 10.3390/nu14194139

**Published:** 2022-10-05

**Authors:** Wenhao Hao, Huali Meng, Hui Li, Yan Zheng, Chunhong Song, Ziping Jiang, Xue Bai, Zhiyue Zhang, Lei Du, Pei Liu, Hao Wu

**Affiliations:** 1 Department of Nutrition and Food Hygiene, School of Public Health, Cheeloo College of Medicine, Shandong University, Jinan 250012, China; 2 Research Center of Translational Medicine, Jinan Central Hospital, Cheeloo College of Medicine, Shandong University, Jinan 250013, China; 3 NMPA Key Laboratory for Technology Research and Evaluation of Drug Products, Key Laboratory of Chemical Biology (Ministry of Education), Department of Pharmaceutics, School of Pharmaceutical Sciences, Cheeloo College of Medicine, Shandong University, Jinan 250012, China; 4 Department of Laboratory Animal Center, Central Hospital Affiliated to Shandong First Medical University, Jinan 250013, China; 5 Department of Hand and Foot Surgery, The First Hospital of Jilin University, Changchun 130021, China; 6 Department of Physical and Chemical Inspection, School of Public Health, Cheeloo College of Medicine, Shandong University, Jinan 250012, China; 7 Department of Plastic Surgery, Qilu Hospital, Cheeloo College of Medicine, Shandong University, Jinan 250012, China

**Keywords:** cutaneous, diabetes, krill oil, skin, wound healing

## Abstract

Diabetes mellitus (DM) impairs the wound healing process, seriously threatening the health of the diabetic population. To date, few effective approaches have been developed for the treatment of diabetic wounds. Krill oil (KO) contains bioactive components that have potent anti-inflammatory and anti-oxidative activities. As prolonged inflammation is a crucial contributor to DM-impaired wound healing, we speculated that the local application of KO would accelerate diabetic wound healing. Therefore, KO was applied to artificially created wounds of type 2 diabetic mice induced by streptozotocin and high-fat diet. The diabetic mice had a delayed wound healing process compared with the non-diabetic control mice, with excessive inflammation, impaired collagen deposition, and depressed neovascularization in the wound area. These effects were dramatically reversed by KO. In vitro, KO blocked the TNF-α-induced macrophage inflammation, fibroblast dysfunction, and endothelial angiogenic impairment. The present study in mice suggests that KO local application could be a viable approach in the management of diabetic wounds.

## 1. Introduction

Diabetes mellitus (DM) is a nutritional disease that seriously threatens public health [1]. Impaired wound healing is a common complication of DM and is the leading cause for wound infection and amputation in the diabetic population [2]. Although hyperbaric oxygen, growth factors, and cytokines have been used for the intervention of DM-impaired wound healing, the outcomes are still unsatisfactory [3]. Hence, the development of new efficient therapeutic approaches is urgently needed.

A series of cells, including macrophages, endothelial cells (ECs), fibroblasts, mesenchymal cells, keratinocytes, and others, orchestrate the normal healing process in an orderly manner [4]. The normal wound healing is generally accomplished in three sequential periods, namely inflammation, proliferation, and remodeling [2]. Under diabetic condition, the fine-tuned periods are disordered. DM leads to a prolonged and excessive inflammatory response that delays wound repair [5]. In diabetic wounds, macrophage infiltration is chronically increased. Moreover, macrophages are prone to be pro-inflammatory M1 polarized, but not anti-inflammatory M2 polarized upon DM [6]. Another hallmark of diabetic non-healing wounds is impaired fiber formation caused by fibroblast dysfunction, which blocks the transforming growth factor beta (TGF-β) profibrotic signaling pathway, hampering collagen accumulation [7,8]. In addition to dysregulated inflammation and fibrosis, DM-depressed neoangiogenesis leads to hypoxia and malnutrition of the wound tissue, thereby contributing to retarded wound healing [9]. These mechanisms, although complicated, provide clues for the intervention of DM-delayed wound repair.

Krill oil (KO) is a functional marine product extracted and purified from Antarctic krill. Rich in docosahexaenoic acid (DHA), eicosapentaenoic acid (EPA), and astaxanthin (AST), KO has strong anti-inflammatory and anti-oxidative activities [10]. We previously found that KO could inhibit DM-induced inflammation and oxidative stress, ameliorating diabetic nephropathy and cardiomyopathy [11,12]. KO has also been reported by other groups to protect against diabetic neuropathy and intestinal inflammation [13,14]. However, the effect of KO on diabetic wound healing has not been previously studied. Given that KO combats inflammation, which is a key mechanism involved in DM-impaired wound healing, we speculate that KO might accelerate diabetic wound healing, at least in part, via its anti-inflammatory activity.

Oral supplementation of DHA and EPA has been reported to benefit wound healing [15]. However, the accumulation of DHA, EPA, and their functional metabolites is limited at the wound site in the form of oral delivery, owing to the loss in the digestion, absorption, and metabolizing procedures. Compared with oral delivery, local application is a more direct way, and is usually more efficient for wound cure [16]. Therefore, KO was studied for its effect on diabetic wound healing in streptozotocin (STZ) and high-fat diet (HFD)-induced type 2 diabetic mice.

## 2. Materials and Methods

### 2.1. Animal Housing and Induction of DM

Seven-week old C57BL/6 male mice were purchased from Jinan Pengyue Laboratory Animal Breeding Co., Ltd. (Jinan, Shandong, China) and were fed in the Animal Center of Shandong University at 22–24 °C, in a 12 h/12 h light/dark cycle with adequate water and food supplies. The Ethics Committee of Preventive Medicine of Shandong University approved all of the experimental procedures (Permission number: SYKX20200022).

Forty mice were culled for the study. To establish DM, after one week of adaptation, the 8-week-old mice received intraperitoneal injection with STZ (Sigma-Aldrich, Shanghai, China) at 50 mg/kg•body weight per day, for five consecutive days. The nondiabetic control (Ctrl) mice were intraperitoneally injected with an equal volume of sodium citrate (0.1 mg/L, pH 4.5) as the vehicle for STZ. One week after the last dose of STZ, the fasting blood glucose levels (6 sh-fast) were measured, with a value above 13.89 mmol/L considered as diabetic. The diabetic or Ctrl mice were then fed an HFD diet or a standard AIN-93G diet, respectively. The blood glucose levels and body weight were monitored every 4 weeks. At the end of the sixth week post DM, a glucose tolerance test (GTT) was performed as previously described [11]. The composition of the experimental diets is listed in Table S1.

### 2.2. Skin Excision and Wound Healing Evaluation

Eight weeks post DM onset, two wounds were made on the upper and lower back (10 mm interval) of each mouse under anesthesia with isoflurane, using a sterile 6 mm skin biopsy punch. The mice were divided into the following groups (10 mice per group): Ctrl (normal wound healing group), DM (diabetic wound without treatment), DM + vehicle (diabetic wound treated with vehicle), and DM + KO (diabetic wound treated with KO dissolved in vehicle). To better preserve the KO in the wounds, KO ointment was prepared by mixing into a vehicle composed of sterile petrolatum (C_15_H_15_N) and paraffin (10:1), forming a concentration of KO at 0.3 mg/g. The KO ointment or the vehicle ointment were applied into the wounds of the diabetic mice once daily. KO was provided by Qingdao Antarctic Weikang Biotechnology Co., Ltd. (Qingdao, Shandong, China). The composition of KO was analyzed and is listed in Table S2.

Images of the wounds were obtained and the diameters were measured on days (D) 0, 4, 7, 10, 13, and 16 after the excision. For each wound, three evenly distributed diameters were recorded. The wound area was calculated using the mean of the diameters of each wound. On D0, D7, and D16, the mice were euthanized and the wounded skin was harvested using a sterile 8 mm skin biopsy punch.

### 2.3. Assessment of Cutaneous Pathology and Immunohistochemical (IHC) Staining

After harvesting, the tissues were fixed into a 10% buffered formalin solution, dehydrated and embedded in paraffin, and sectioned into 5 µm thick sections onto glass slides for staining. Hematoxylin and eosin (H&E) staining (ThermoFisher Scientific, Shanghai, China) was used to evaluate the cutaneous morphology, wound area cutaneous thickness, and number of follicles. Cutaneous collagen accumulation was assessed by Masson’s trichrome staining (Solarbio, Beijing, China). Image J (National Institutes of Health, Bethesda, MD, USA) was used for quantification of the cutaneous thickness and Masson’s positive area. The selection of the imaging area and scoring were performed by people who were blind to the identity of the samples.

IHC staining was carried out as previously described [11], using primary antibodies against CD31 (1:200, Proteintech, Wuhan, Hubei, China) and F4/80 (1:400, Cell Signaling Technology, Shanghai, China).

### 2.4. Cell Culture and Treatments

To study the effect of KO on macrophage polarization, fibroblast function, and neoangiogenesis, RAW 264.7 cells (cultured in Dulbecco’s modified Eagle’s medium (DMEM), containing 10% fetal bovine serum (FBS, ThermoFisher Scientific, Shanghai, China)), L929 cells (Cultured in DMEM containing 5% FBS), and human vascular ECs (Cultured in endothelial cell medium containing 10% FBS, ScienCell, Beijing, China) were pretreated with KO (0.25 mg/mL) or the vehicle glycerol (GLY, 25 mg/mL, Sinopharm Chemical Reagent Co., Ltd., Shanghai, China) for 24 h, and were then stimulated with TNF-α for 6 h.

### 2.5. Cell Scratch Assay

To evaluate the effect of KO on fibroblast migration, the KO (0.25 mg/mL)- or GLY (25 mg/mL)-pretreated L929 cells were stimulated with TNF-α (10 ng/mL) for 6 h. A scratch was then made using the bottom of a 10 µL sterile pipette tip in each well of cells. Closure of the wounds was monitored by photographing 0, 12, and 18 h after the scratches, and the cell migration widths were calculated.

### 2.6. Matrigel Tube Formation Assay

A matrigel tube formation assay was performed to investigate whether KO could enhance angiogenesis. Briefly, ECs were pretreated with KO (0.25 mg/mL) or GLY (25 mg/mL) for 24 h, followed by challenging with TNF-α (10 ng/mL) for 6 h. Pre-cooled matrigel was plated in a 96-well plate on ice, and then gelled in an incubator at 37 °C for 30 min. The cells were digested, suspended, and plated into the 96-well plate and cultured at 37 °C. Then, 6 h later, tube formation and disruption were observed and photographed.

### 2.7. Cell Immunofluorescence

For cell immunofluorescence, ECs were seeded in a 24-well plate. After pretreating with KO (0.25 mg/mL) or GLY (25 mg/mL) for 24 h, the cells were stimulated with TNF-α (10 ng/mL) for 6 h. After discarding the medium, the cells were washed with PBS three times, then blocked with 10% formaldehyde for 15 min. After washing with PBS three times, the cells were blocked with 5% bovine serum albumin for 1 h. The cells were then incubated with a primary antibody against E-cadherin (1:400, Proteintech, Wuhan, China) at 4 °C overnight, followed by washing with PBS three times and incubating with a secondary antibody for 1 h in the dark. After washing with PBS three times, the fluorescence was observed and captured using a fluorescence microscope.

### 2.8. RNA Extraction and Quantitative Real-Time Polymerase Chain Reaction (qRT-PCR)

The cutaneous and cellular RNA was extracted and underwent qRT-PCR analysis for determining the RNA levels, using primers for actin alpha 2 (*Actα2*), angiopoietin-2 (*Angpt2*), angiopoietin-4 (*Angpt4*), collagen type I alpha 1 (*Col1α1*), *E-CADHERIN*, fibronectin (*Fn*), Interleukin-10 *(Il-10*), interleukin 1 beta (*Il-1β*), matrix metalloproteinase1 (*Mmp1*), matrix metalloproteinase 1 (*Mmp2*), nitric oxide synthase 2 (*Nos2*), *Rplp0*, *Serpine1*, *Tgf-β1*, vascular cell adhesion molecule 1 (*Vcam1*), and vascular endothelial growth factor (*VEGF-A*, Sangon Biotech, Shanghai, China). The procedures for RNA extraction and qRT-PCR were described previously [12]. The sequences of the primers are provided in Table S3.

### 2.9. Western Blot Analysis

Western blot was performed to measure the protein levels using cutaneous tissue and cell lysates, as previously described [12], using primary antibodies against alpha-smooth muscle actin (α-SMA, 1:1000, Santa Cruz Biotechnology, Shanghai, China), β-tubulin (1:1000, Proteintech), E-cadherin (1:1000, Proteintech), GAPDH (1:10000, Proteintech), IL-1β (1:1000, Cell Signaling Technology, Shanghai, China), NF-κB (1:1000, Cell Signaling Technology), *p*-NF-κB (1:1000, Cell Signaling Technology), TGF-β1 (1:1000, Cell Signaling Technology), VCAM1 (1:1000, Abcam, Shanghai, China), and VEGF-A (1:1000, ABclonal, Wuhan, Hubei, China). Western blot images were quantified utilizing Image StudioTM Lite software (LI-COR, Lincoln, NE, USA).

## 3. Results

### 3.1. KO Accelerated Wound Healing in the Diabetic Mice

**Figure 1:**
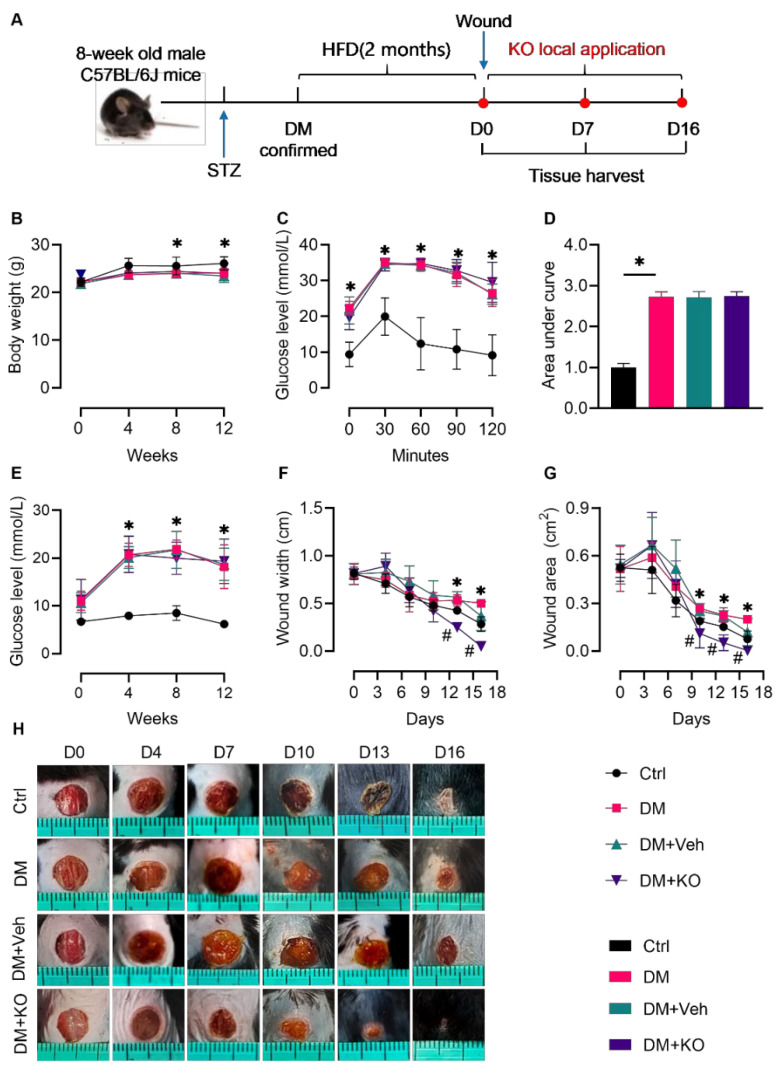
KO accelerated wound healing in the diabetic mice. (**A**) Schematic diagram for the induction of DM and the cutaneous wound, and the local application of KO. (**B**) Body weight. (**C**) GTT assay with the (**D**) area under the blood glucose curve quantified. (**E**) Fasting blood glucose levels post the onset of DM. (F) Wound width and (**G**) area quantified from (**H**) the images of the wounds. The data were summarized as means ± SD. *, *p* < 0.05 vs. Ctrl; #, *p* < 0.05 vs. DM. Ctrl, control; D, day; DM, diabetes mellitus; GTT, glucose tolerance test; HFD, high-fat diet; KO, krill oil; STZ, streptozotocin.

To assess the effect of KO on wound healing in the diabetic mice, KO was locally applied into the wounds on the backs of the diabetic mice induced by STZ and HFD (Figure 1A). KO did not change the body weight, which was decreased in the diabetic mice (Figure 1B). The diabetic mice developed glucose intolerance (Figure 1C,D) and increased fasting blood glucose levels (Figure 1E), the effects of which were not affected by KO (Figure 1C–E). Notably, local KO application significantly accelerated the diabetic wound healing as reflected by the decreased wound width (Figure 1F; D13 and D16) and area (Figure 1G,H; D10, D13, and D16).

**Figure 2:**
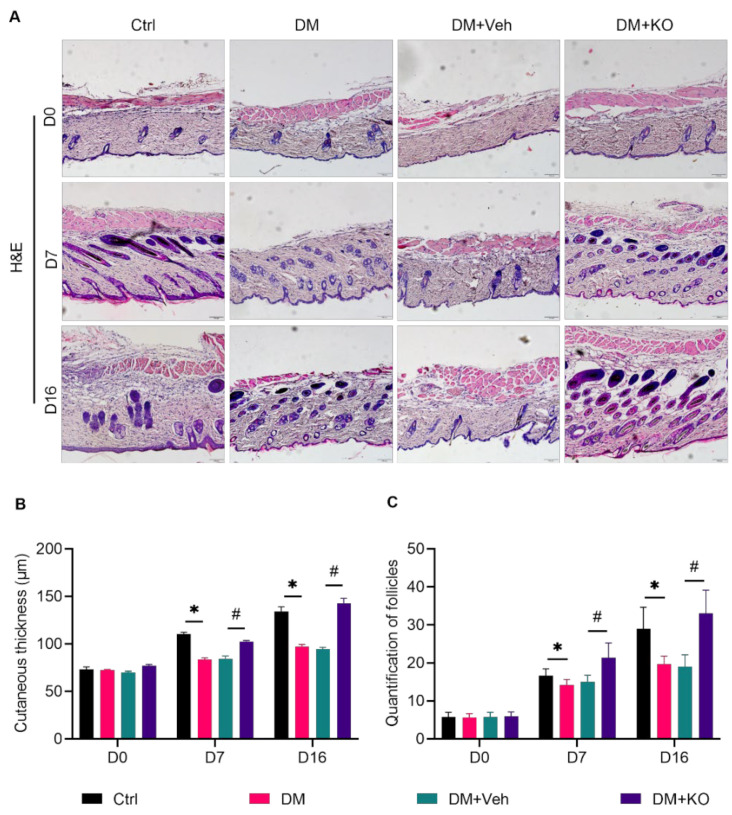
KO accelerated wound healing in the diabetic mice. (**A**) H&E staining was performed to assess (**B**) the cutaneous thickness and (**C**) follicle number of the wounds. The data were summarized as means ± SD. *, *p* < 0.05 vs. Ctrl; #, *p* < 0.05 vs. DM. Abbreviations are the same as in Figure 1.

H&E staining (Figure 2A) was performed to determine the cutaneous thickness (Figure 2B) and follicle number (Figure 2C) at the wound area as an alternative evaluation of the wound healing. DM delayed the cutaneous thickening and follicle formation on D7 and D16 post excision (Figure 2B,C). These effects were reversed by KO (Figure 2B,C).

### 3.2. KO Attenuated the DM-Induced Cutaneous Inflammation

**Figure 3:**
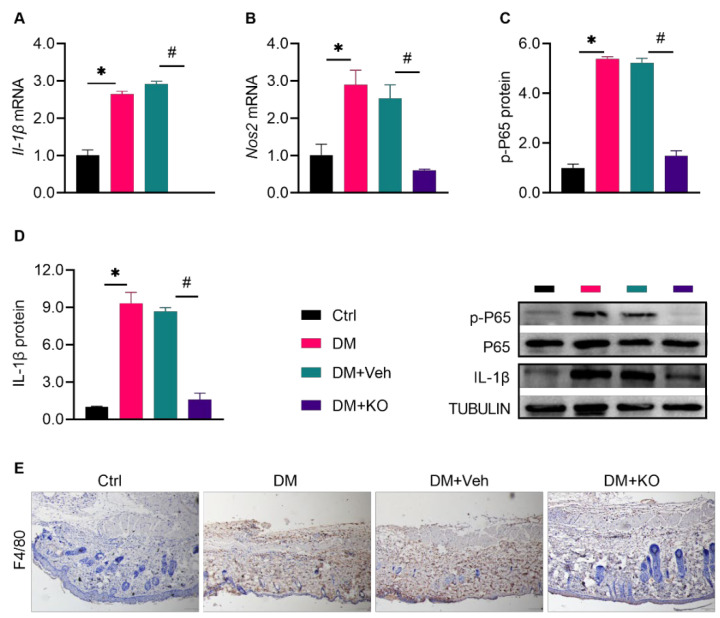
KO attenuated the DM-induced cutaneous inflammation. Seven days post skin excision, the (**A**) *Il-1β* mRNA levels, (**B**) *Nos2* mRNA levels, (**C**) protein ratio of *p*-P65 to P65, (**D**) IL-1β protein levels were determined, and (**E**) IHC staining was performed to detect the expression of the macrophage marker F4/80 in the wound tissue. For (**A**–**D**), the data were normalized to Ctrl, and summarized as means ± SD. *, *p* < 0.05 vs. Ctrl; #, *p* < 0.05 vs. DM. IHC, immunohistochemical; *Il-1β*, interleukin 1 beta; *Nos2*, nitric oxide synthase 2. The other abbreviations are the same as in Figure 1.

Sustained inflammation delays the healing process of diabetic wounds [17]. DM resulted in an enhanced expression of cutaneous *Il-1β* mRNA, *Nos2* mRNA, protein ratio of *p*-P65 to P65, and IL-1β protein in the wound area (Figure 3A–D). Macrophage infiltration was also increased in the diabetic wound, as shown by F4/80 staining (Figure 3E). These effects were abolished by KO (Figure 3A–E).

### 3.3. KO Alleviated the TNF-α-Induced Macrophage Inflammation

**Figure 4:**
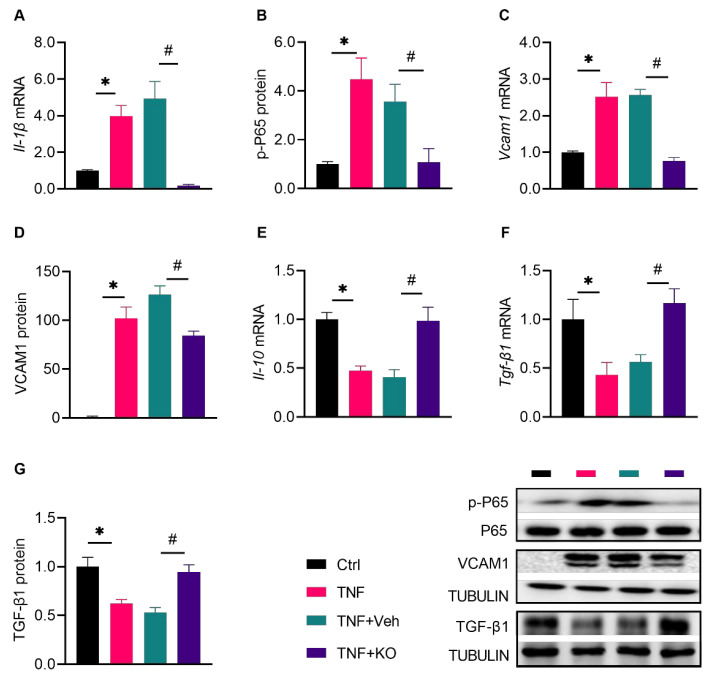
KO alleviated the TNF-α-induced macrophage inflammation. (**A**) *Il-1β* mRNA levels, (**B**) protein ratio of *p*-P65 to P65, (**C**) *Vcam1* mRNA levels, (**D**) VCAM1 protein levels, and (**E**) *Il-10* mRNA levels, as well as (**F**) *Tgf -β1* mRNA and (**G**) protein levels were determined. The data were normalized to Ctrl, and summarized as means ± SD. *, *p* < 0.05 vs. Ctrl; #, *p* < 0.05 vs. DM. *Il-10*, Interleukin-10; TGF-β1, transforming growth factor β 1; VCAM1, vascular cell adhesion molecule 1. The other abbreviations are the same as in Figure 3.

M1 polarization of macrophages is a major cause for excessive inflammation during diabetic wound healing [18]. The TNF-α level is increased in diabetic wounds, provoking macrophage M1 polarization [19]. In RAW264.7 cells, KO reduced the expression of the TNF-α-induced expression of M1 markers such as *Il-1β* mRNA, the *p*-P65 to P65 ratio, and *Vcam1* mRNA and protein (Figure 4A–D), and reversed the TNF-α-prohibited expression of *Il-10* and *Tgf-β1* as markers for M2 polarization (Figure 4E–G).

### 3.4. KO Facilitated Cutaneous Collagen Deposition in the Diabetic Wounds

**Figure 5:**
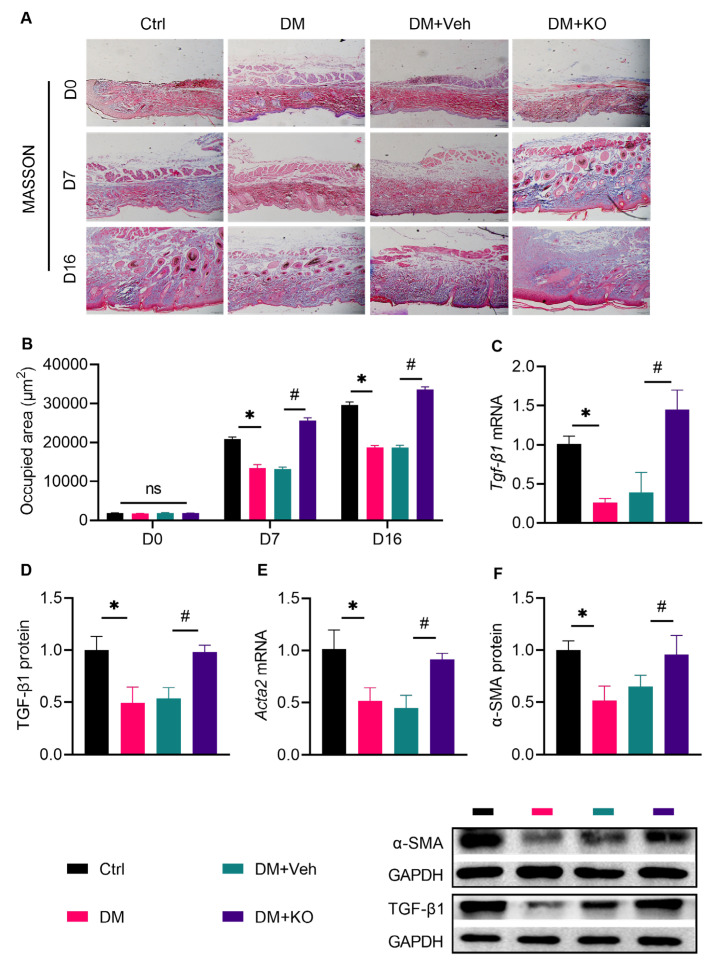
KO facilitated cutaneous collagen deposition in the diabetic wounds. (**A**) Masson’s trichrome staining was performed to assess (**B**) the deposition of mature fibers on D0, D7 and D16. (**C**) *Tgf-β1* mRNA levels, (**D**) TGF-β1 protein levels, (**E**) *Acta2* mRNA levels, and (**F**) α-SMA protein levels were determined. The data were summarized as means ± SD. *, *p* < 0.05 vs. Ctrl; #, *p* < 0.05 vs. DM. *Actα2*, actin α 2; α-SMA, α-smooth muscle actin. The other abbreviations are the same as in Figure 1 and Figure 4.

Collagen deposition is essential for normal wound healing, as collagen supports functional cells for wound healing and shrinks the wounds [20]. Masson’s trichrome staining (Figure 5A) showed that collagen accumulation was impaired in the diabetic mice on D7 and D16 (Figure 5B). KO significantly accelerated this process (Figure 5B). TGF-β1 and α-SMA are sensitive factors for fibroblast differentiation, leading to collagen formation [21]. KO reactivated the cutaneous expression of *Tgf-β1* and *Acta2*, both of which were inhibited under DM (Figure 5C–F).

### 3.5. KO Promoted the Profibrotic Transition of TNF-α-Stimulated Fibroblasts

**Figure 6:**
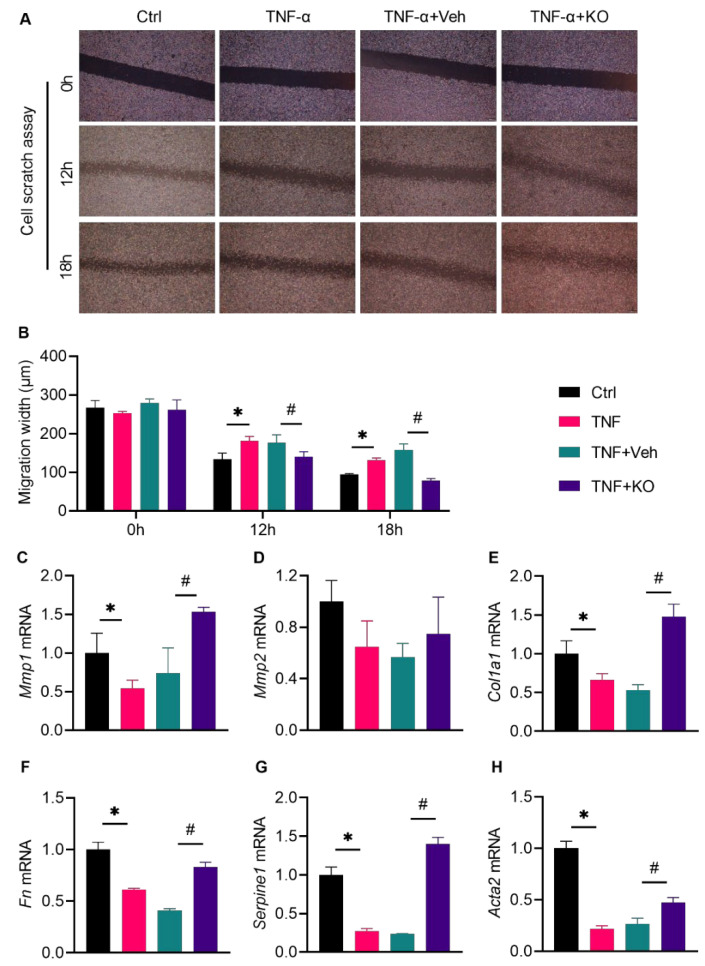
KO promoted the profibrotic transition of the TNF-α-stimulated fibroblasts. (**A**) A cell scratch assay was performed to assess the migrating activity of L929 cells treated with TNF-α, with the (**B**) migration width measured and quantified. (**C**) The *Mmp1* mRNA levels, (**D**) *Mmp2* mRNA levels, (**E**) *Col1a1* mRNA levels, (**F**) *Fn* mRNA levels, (**G**) *Serpine1* mRNA levels, and (**H**) *Acta2* mRNA levels were determined. The data were summarized as means ± SD. *, *p* < 0.05 vs. Ctrl; #, *p* < 0.05 vs. DM. *Col1α1*, collagen 1 α 1; *Fn*, fibronectin; *Mmp1*, matrix metalloproteinase 1; *Mmp2*, matrix metalloproteinase 2; *Serpine1*, serine (or cysteine) peptidase inhibitor 1. The other abbreviations are the same as in Figure 5.

Cutaneous fibroblasts are major functional cells during wound healing, owing to their proliferative, migrating, and extracellular matrix-producing activities [22]. The scratch migration assay (Figure 6A) identified an impairment in the fibroblast migrating ability upon TNF-α treatment for 12 and 18 h (Figure 6B). These effects were abrogated by KO, as revealed by the decreased migration width (Figure 6B). Further investigation showed that KO activated the mRNA expression of the profibrotic transition genes *Mmp1*, *Col1a1*, *Fn*, *Serpine1,* and *Acta2* in TNF-α-stimulated fibroblasts, expect for *Mmp2* (Figure 6C–H).

### 3.6. KO Enhanced Neoangiogenesis in Diabetic Wounds

**Figure 7:**
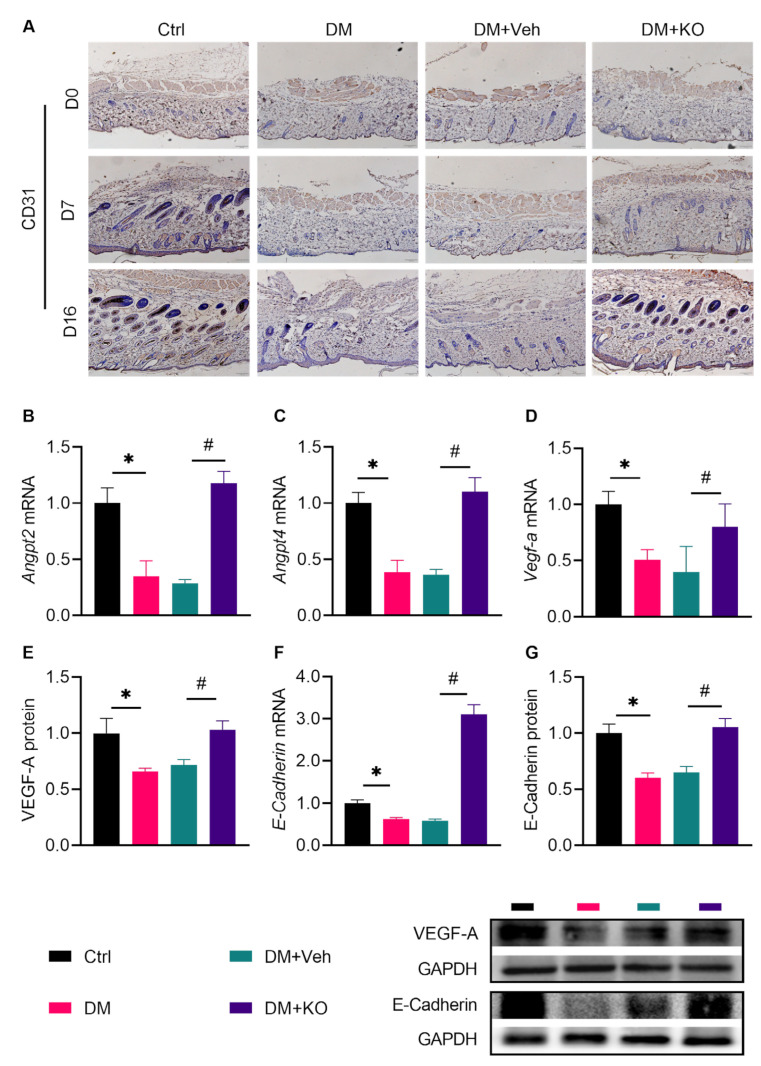
KO enhanced neoangiogenesis in the diabetic wounds. (**A**) IHC staining of CD31. The (**B**) *Angpt2* mRNA levels, (**C**) *Angpt4* mRNA levels, (**D**) *Vegf-a* mRNA and (**E**) protein levels, (**F**) *E-Cadherin* mRNA, and (**G**) protein levels were determined. The data were summarized as means ± SD. *, *p* < 0.05 vs. Ctrl; #, *p* < 0.05 vs. DM. *Angpt2*, Angiopoietin-2; *Angpt4*, Angiopoietin-4; VEGF-A, Vascular Endothelial Growth Factor A. The other abbreviations are the same as in Figure 3 and Figure 6.

Neovascularization is another key mechanism involved in wound healing [23]. CD31, a marker for ECs, was increased in the wound area by KO (Figure 7A). In addition, KO dramatically activated the cutaneous expression of *Angpt2* mRNA, *Angpt4* mRNA, *Vegf-a* mRNA, and protein, as well as *E-Cadherin* mRNA and protein (Figure 7B–G), confirming that KO enhanced neoangiogenesis in the diabetic wounds.

### 3.7. KO Induced Angiogenesis in TNF-α-Treated ECs

**Figure 8:**
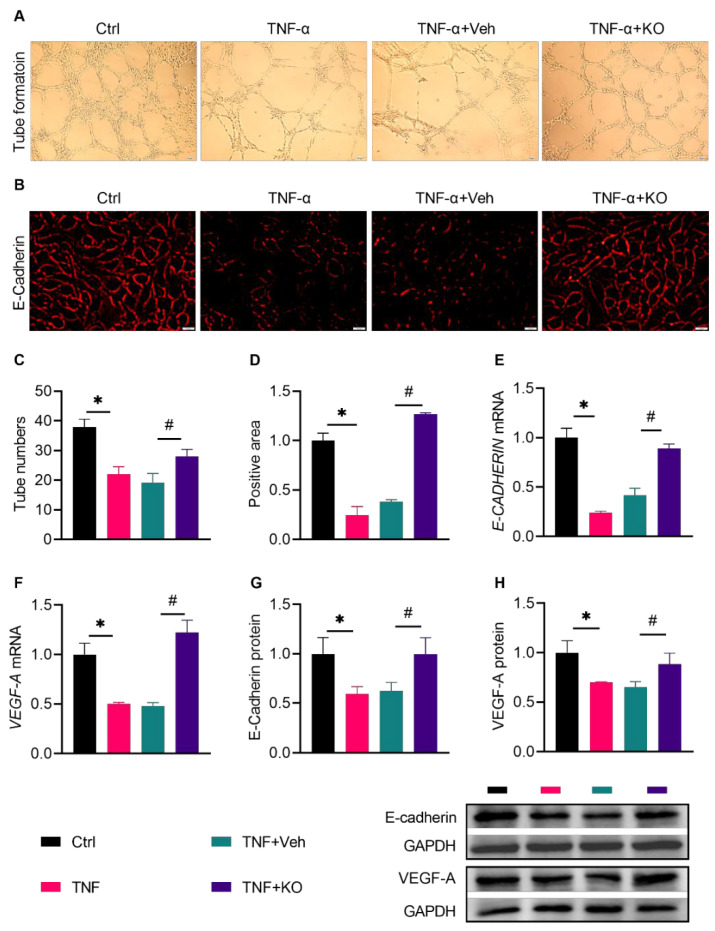
KO induced angiogenesis in TNF-treated ECs. (**A**) The matrigel tube formation assay and (**B**) immunofluorescent staining of E-Cadherin were performed to verify the effect of KO on angiogenesis in TNF-α-treated ECs. (**C**) The number of tube formation. (**D**) E-Cadherin positive area quantified from the immunofluorescence. (**E**) *E-CADHERIN* mRNA levels. (**F**) *VEGF-A* mRNA levels. Protein levels of (**G**) E-Cadherin and (**H**) VEGF-A. The data were summarized as means ± SD. *, *p* < 0.05 vs. Ctrl; #, *p* < 0.05 vs. DM. The abbreviations are the same as in Figure 7.

In order to further verify the effect of KO on angiogenesis in vitro, KO was tested for its effect on EC tube formation and E-CADHERIN expression (Figure 8A,B). EC tube formation was impaired upon TNF-α stimulation and was rescued by KO (Figure 8C). Moreover, KO reversed the TNF-α-reduced expression of *E-Cadherin*, a marker for ECs, as shown by the immunofluorescent staining (Figure 8D). qRT-PCR and Western blot further confirmed that KO activated the expression of *E-CADHERIN* and *VEGF-A,* which were inhibited by TNF-α (Figure 8E–H), demonstrating that KO activated angiogenesis in vitro.

## 4. Discussion

**Figure 9:**
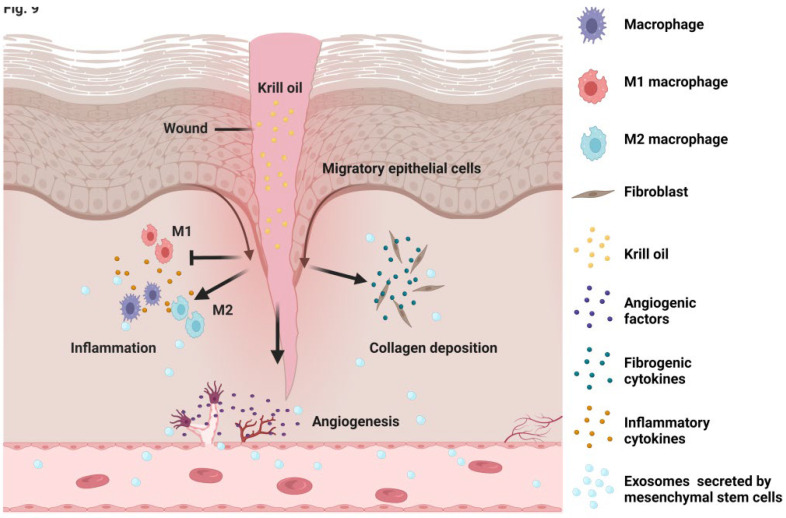
Schematic diagram for the actions of KO on diabetic wound healing. Macrophage, fibroblasts, and endothelial cells participate in the effect of KO on diabetic wound healing. Specifically, KO induced the M1 to M2 polarization of macrophages, increased the migration and collagen production of fibroblasts, and enhanced the neovascularization of endothelial cells.

The present study found that local KO application significantly accelerated wound healing in a mouse model of type 2 DM (T2DM). Mechanistically, KO controlled the cutaneous excessive inflammation, enhanced collagen production, and promoted neovascularization in both the diabetic wounds and the TNF-α-challenged cells (Figure 9). To date, this has been the first report regarding the effect of KO on wound healing.

Inflammation mostly occurs in the early stage of wound healing. In normal conditions, the inflammation period is usually short in time and strong in extent, recruiting immune cells for self-defense. This is beneficial for infection prevention and wound repair [24]. Under the diabetic condition, the inflammation period is prolonged and sustained, delaying wound healing [23]. Compared with inflammation, fibrosis and neoangiogenesis occur at a relatively later stage [25,26], and are inhibited upon DM [26]. In the present study, DM was found to induce a prominent inflammatory state in D7 and to inhibit fibrosis and neoangiogenesis in D16 (Figure 3, Figure 5, and Figure 7), confirming the temporal specificity of different pathophysiological processes in diabetic wound healing. Awareness of the temporal pathophysiological specificities may be helpful for designing targeted approaches for the intervention of diabetic non-healing wounds.

KO has been reported to have anti-inflammatory, anti-oxidative, and anti-fibrotic activities in mouse models of diabetic complications [11,12,27]. We previously found that KO could inhibit DM-induced cardiac and renal inflammation and fibrosis, preventing diabetic cardiomyopathy and nephropathy in a mouse model of T2DM [11,12]. In line with these findings, in the present study, KO was found to attenuate cutaneous inflammation in the diabetic wounds (Figure 3). However, different from the anti-fibrotic effect of KO on the diabetic hearts and kidneys, KO generated cutaneous fibrosis in the diabetic mice (Figure 5). In particular, KO activated the cutaneous expression of TGF-β1, α-SMA, and collagen as the initiator, intermediate product, and end product of fibrosis (Figure 5), respectively, whereas TGF-β1 signaling and the expression of α-SMA and collagen were significantly inhibited by KO in the diabetic hearts and kidneys [11,12]. The discrepancy between the effects of KO on fibrosis in different tissues might be attributed to the different cell identities during the pathogenesis of diabetic complications. In response to the diabetic condition, cells are mostly pro-fibrotic in the heart and kidney, but are inefficient to generate fibrosis in the cutaneous wound. Furthermore, DM resulted in prolonged inflammation, which contributes to impaired cutaneous fibrosis, but enhances cardiac and renal profibrotic actions [28]. The double-edged effects of inflammation on fibrosis in different organs under DM might be an explanation for the different actions of KO on fibrosis between the skin and the heart/kidney, although further studies may be needed to explore the molecular actions of KO in different tissues.

In the present work, macrophages, fibroblasts, and ECs were culled for the study of KO’s effects in vitro. As major functional cells in cutaneous inflammation, fibrosis. and neoangiogenesis, these cells may mimic the in vivo biological processes during wound healing. TNF-α is increased in diabetic wounds [29]. In the present study, TNF-α induced macrophage M1 polarization, blunted macrophage M2 polarization, induced fibroblast dysfunction, and restrained EC angiogenesis (Figure 4, Figure 6, and Figure 8). These findings indicate that TNF-α is a valid stimulus for the study of diabetic wound healing in these cell types. In addition to macrophages, fibroblasts, and ECs, remote regulatory cells such as mesenchymal stem cells (MSCs) or their derivatives exert paracrine functions, modulating cutaneous inflammation, fibroblast activation, neovascularization, and re-epithelialization, which play key roles in skin regeneration [30]. This might be partially achieved by the MSC exosomes-induced transition of M1 to M2 polarization of macrophages [31]. Moreover, treatment with MSCs-excreted exosomes improved the survival, migration, and extracellular matrix production of fibroblasts isolated from the wounds of both diabetic and non-diabetic individuals, suggesting a direct action of MSC exosomes on fibroblast function [32]. MSCs also secrete angiopoietin 1 (Ang1), Ang2, and VEGF, which enhance vascular stability [31,32,33]. The findings from the present work and other studies suggest that targeting the functional cells involved in wound healing is an efficient strategy to accelerate wound repair.

Containing DHA, EPA, AST, and other ingredients, KO must have exerted its effects on diabetic wound healing through multiple mechanisms. DHA, EPA, and AST, as the major functional components of KO, are known to benefit wound repair. DHA was reported to activate the expression of VEGF to facilitate wound healing in STZ-induced diabetic mice [34]. EPA promoted endothelial progenitor cell migration-mediated neovascularization [35]. In lipopolysaccharide-stimulated fibroblasts, AST eliminated ROS and inflammation, and improved the cell viability and proliferative capacity [36]. However, it is still unknown whether these components can yield an additive or synergistic effect on wound healing. It would thus be interesting to compare the effect of DHA, EPA, AST, and KO in future studies.

One notable innovation of the present study is the local delivery of KO to wounds. For wound management, local application has several advantages over oral delivery. Local application allows for a high concentration of KO in the wound, ensuring therapeutic effects. Additionally, the local application of KO avoids waste during bioavailable processes such as digestion, absorption, and metabolizing, which can reduce therapeutic costs. Antarctic krill is an abundant natural resource, and the extraction and manufacturing process of KO is technically mature. Considering the availability of KO and its profound effect on diabetic wound healing, the present study presents the local application of KO as a potential novel, efficient, inexpensive, and practical therapeutic approach for the future clinical intervention of diabetic wounds.

The present work has advantages and weaknesses. By using a mouse and three cell models, the present study provides a systematic analysis of the effect of KO on diabetic wound healing, revealing multiple molecular actions of KO in facilitating wound repair. Additionally, the local application of KO was demonstrated by the present study to be an efficient method for the delivery of bioactive food components to diabetic wounds. However, the roles of specific components within KO were not further investigated in this work. These need to be explored in future studies.

## 5. Conclusions

The present study found a dramatic accelerating effect of local KO application on diabetic wound healing in mice, providing a basis for the future application of this method of treatment for the management of wound healing in diabetic individuals.

## Data Availability

The datasets used and/or analyzed during the current study are available from the corresponding author upon reasonable request.

## Supplementary Materials

The following are available online at https://www.mdpi.com/article/10.3390/nu14194139/s1. Table S1. Ingredients composition of experimental diets. Table S2. Composition analysis of krill oil used in the study. Table S3. Sequences of primers used for qRT-PCR.
