# Supplementary material for: Local Application of Krill Oil Accelerates the Healing of Artificially Created Wounds in Diabetic Mice
Source: Nutrients. 2022 Oct 5;14(19):4139. doi: 10.3390/nu14194139 (PMC9571309; doi:10.3390/nu14194139)
Supplement: Supplementary file 1 [file nutrients-14-04139-s001.zip › nutrients-1941991-supplementary.pdf]

Table S1. Ingredients composition of experimental diets

Ingredient (g/kg)	Normal diet	High-fat diet
Soybean oil	70	70
Krill oil	-	-
Lard	-	230
β-cornstarch	397.49	110.62
α-(dextrinized) cornstarch	132	36.87
Casein	200	250
Sucrose	100	200
AIN-93 mineral mixture	35	35
AIN-93 vitamin mixture	10	10
L-cystine	3	3.75
Choline bitatrate	2.5	2.5
Cellulose	50	50
Tert-Butylhydroquinone	0.01	0.06
Cholesterol	-	1.2
Protein	203	253.75
Carbohydrates	629.49	347.49
Fat	70	300

Table S2. Composition analysis of krill oil

Krill oil	Result	Unit
Astaxanthin	232.15	mg/kg
Peroxide	< 0.2	eEq/kg
Saponification	163.0	mg/KOH/g
Total phospholipids	55.06	g/100g
Fatty acid composition		%/total fatty acids
C14:0	10.51	
C16:0	25.77	
C16:1	6.15	
C18:0	0.28	
C18:1	14.2	
C18:2	1.33	
C18:3	1.59	
C20:3	1.19	
C20:4	0.73	
C20:5 (EPA)	22.89	
C22:6 (DHA)	13.83	

Table S3. Sequences of primers used for qRT-PCR

Genes	Sequences
Actα2-forward	TAACCCTTCAGCGTTCAGC
Actα2-reverse	ACATAGCTGGAGCAGCGTCT
Angpt2-forward	ACATGAAGAAGGAGATGGTG
Angpt2-reverse	CGTCTGGTTTAGTACTTGGG
Angpt4-forward	TCCTTAAAGACACCTAAGCCAGTG
Angpt4-reverse	GGTCCTCTGGAAATTTACGCTTCC
Col1α1-forward	CCGCTGGTCAAGATGGTC
Col1α1-reverse	CTCCAGCCTTTCCAGGTTCT
E-cadherin-forward	CGAGAGAGTTACCCTACATA
E-cadherin-reverse	GTGTTGGGGGCATCATCATC
E-CADHERIN-forward	GTCTGTAGGAAGGCACAGCC
E-CADHERIN-reverse	TCATCCTCTGGGGGCAGTAA
Fn1-forward	CGGAGAGAGTGCCCCTACTA
Fn1-reverse	CGATATTGGTGAATCGCAGA
Il-10-forward	GGCAGAGAACCATGGCCCAGAA
Il-10-reverse	AATCGATGACAGCGCCTCAGCC
Il-1β-forward	AAATGCCACCTTTTGACAGTGATG
Il-1β-reverse	GCAGCCCTTCATCTTTTGGG
Mmp1-forward	CCCACAATGTCCCCATCTATG
Mmp1-reverse	TGAACAGCCCAGTACTTATTCC
Mmp2-forward	CCATTTTGATGACGATGAGCTATG
Mmp2-reverse	GTTGTACTCCTTGCCATTGAACAA

Nos2-forward	TGCCTCTCACTCTTCCTTGG
Nos2-reverse	CCCAAAGTGCTTCAGTCAGG
Rplp0-forward	AGCAAAGGAAGAGTCGGAGG
Rplp0-reverse	GGCTGACTTGGTTGCTTTGG
Serpine1-forward	AGGATCGAGGTAAACGAGAGC
Serpine1-reverse	GCGGGCTGAGATGACAAA
Tgf-β1-forward	AGGAAGGACCTGGGTTGGAAG
Tgf-β1-reverse	CGTCTCGACCCACGTAGTAGACG
Vcam1-forward	TCTTACCTGTGCGCTGTGAC
Vcam1-reverse	ACTGGATGTTCAGGGAATGAGT
Vegf-a-forward	CTGCCGTCCGATTGAGACC
Vegf-a-reverse	CCCCTCCTTGTACCACTGTC
VEGF-A-forward	TTGCAGGTTGGTTCCCAGAGG
VEGF-A-reverse	TCGGCTTGTCACATCTGAGGG
